# Langjährige Myalgien und Hypourikämie bei einer jungen Frau: Fallbeschreibung und Review der Literatur

**DOI:** 10.1007/s00393-025-01689-x

**Published:** 2025-08-14

**Authors:** Ruta Venyte, Corina Schuster-Amft, Frank Behrendt, Nicole Nyfeler, Katrin Parmar, Johannes Lorscheider, Leo H. Bonati, Ulrich A. Walker, Hans Ulrich Gerth

**Affiliations:** 1 Research Department, Reha Rheinfelden, Salinenstr. 98, 4310 Rheinfelden, Schweiz; 2 School of Engineering and Computer Science, Bern University of Applied Sciences, Biel, Schweiz; 3 Department for Sport, Exercise and Health, University of Basel, Basel, Schweiz; 4 Translational Imaging in Neurology (ThINk) Basel, Departments of Head, Spine and Neuromedicine and Biomedical Engineering, University Hospital Basel and University of Basel, Basel, Schweiz; 5 Neurologic Clinic and Policlinic, MS Center and Research Center for Clinical Neuroimmunology and Neuroscience Basel (RC2NB), University Hospital Basel and University of Basel, Basel, Schweiz; 6 Stroke Center and Department of Neurology, University Hospital Basel, Basel, Schweiz; 7 Department of Clinical Research, University of Basel, Basel, Schweiz; 8 Department of Rheumatology, University Hospital Basel, Basel, Basel-Stadt Schweiz; 9 Laboratory for Experimental Rheumatology, Department of Biomedicine, University of Basel, Basel, Basel-Stadt Schweiz; 10 Department of Medicine, University Hospital Münster, Münster, Deutschland

**Keywords:** Fibromyalgie, Myalgie, Hypourikämie, Xanthinurie, Fibromyalgia, Myalgia, Hypouricemia, Xanthinuria

## Abstract

Eine 46-jährige Patientin leidet seit dem 27. Lebensjahr an multiplen Symptomen wie Arthralgien, Myalgien, allgemeiner Müdigkeit, Erschöpfung, Konzentrationsstörungen, Vergesslichkeit, Ein- sowie Durchschlafstörungen und Depression. Rheumatologisch wurden vorbefundlich eine rheumatoide Arthritis diagnostiziert mit fehlendem Ansprechen auf eine Basistherapie sowie ebenfalls eine sekundäre Fibromyalgie. Bei laborchemisch Hypourikämie mit nicht nachweisbarer Harnsäure im Serum erfolgten ergänzende Stoffwechseluntersuchungen, die massiv erhöhte Xanthinwerte im Urin zeigten. Bei normwertiger Nierenfunktion und unauffälligem Urinsediment konnten sonographisch kleine Konkremente im Nierenbeckenkelchsystem beidseits dargestellt werden. Es konnte die Diagnose einer Xanthinurie gestellt werden. Hierbei handelt es sich um eine seltene autosomal-rezessiv vererbte Störung des Purinstoffwechsels mit fehlender Umwandlung von Hypoxanthin über Xanthin zu Harnsäure über das Enzym Xanthinoxidoreduktase. Klinische Verläufe sind teilweise asymptomatisch bis zu symptomatischer Urolithiasis bei erhöhter renaler Xanthinausscheidung und Myalgien bei intramuskulären Xanthinablagerungen. Zudem werden unspezifische Arthralgien und eine progrediente Niereninsuffizienz im Verlauf der Erkrankung beschrieben. Klinisch kann bei der Symptomüberlappung der Fibromyalgie und Xanthinurie nicht sicher zwischen beiden Entitäten differenziert werden.

**Zusatzmaterial online:**

Die Online-Version dieses Beitrags (10.1007/s00393-025-01689-x) enthält weiterführende Informationen.

## Abstract (en)

A 46-year-old female patient had been suffering from multiple symptoms such as arthralgia, myalgia, general fatigue, exhaustion, concentration problems, forgetfulness, difficulty falling asleep and sleeping through the night and depression since the age of 27 years old. Rheumatological preliminary findings revealed rheumatoid arthritis with a lack of response to basic treatment as well as secondary fibromyalgia. Supplementary metabolic examinations were carried out in the case of laboratory tests for hypouricemia, which showed massively increased xanthine levels in the urine. With normal renal function and unremarkable urine sediment, small concrements were sonographically visualized in the renal pelvic calyceal system on both sides. The diagnosis of xanthinuria was made. This is a rare hereditary disorder of purine metabolism with a lack of conversion of hypoxanthine via xanthine to uric acid via the enzyme xanthine oxidoreductase. Clinical courses range from asymptomatic to symptomatic urolithiasis with increased renal xanthine excretion and myalgia with intramuscular xanthine deposits. In addition, non-specific arthralgia and progressive renal insufficiency are described during the course of the disease. Clinically, the overlapping symptoms of fibromyalgia and xanthinuria make it impossible to reliably differentiate between the two entities.

## Anamnese

Eine 46-jährige Patientin leidet seit dem 27. Lebensjahr an Arthralgien, Myalgien, allgemeiner Müdigkeit, Erschöpfung, Konzentrationsstörungen, Vergesslichkeit, Ein- sowie Durchschlafstörungen und Depression. Im Jahr 2020 wurde eine seronegative rheumatoide Arthritis diagnostiziert bei sonographisch Ergüssen in multiplen Kleinfingergelenken beidseits mit Power-Doppler-Aktivität. Eine Basistherapie mit Methotrexat und Prednison wurde begonnen und nach wenigen Monaten bei fehlender Wirkung auf die Arthralgien sowie gastrointestinaler Symptomatik beendet. Die Arthritiden rezidivierten im Verlauf auch nicht, sodass retrospektiv die Diagnose der rheumatoiden Arthritis infrage gestellt werden kann. Im Verlauf wurde die Diagnose einer sekundären Fibromyalgie bei Druckschmerzhaftigkeit aller ACR(American College of Rheumatology)-Tenderpoints (18/18) in der klinischen Untersuchung gestellt. Alle multimodalen Therapieversuche (Homöopathie, Akupunktur, Schröpfen, Physiotherapie, Wassertherapie, medizinische Trainingstherapie, Analgetika) brachten keine dauerhafte Linderung.

## Befund

Die Patientin stellte sich in der rheumatologischen Sprechstunde zur Zweitmeinung in sehr gutem Allgemeinzustand und normalem Ernährungszustand vor. Der rheumatologische Status war insgesamt unauffällig bis auf eine diffuse Berührungsempfindlichkeit der Haut, eine allgemeine nicht gelenkbezogene Druckschmerzhaftigkeit sowie paravertebrale Myogelosen. Insbesondere zeigten sich keine Arthritis und eine unauffällige Textur der Muskulatur ohne Vaskulitis- oder Kollagenosestigmata. Vorbefundlich dokumentiert sind negative Rheumafaktoren, antinukleäre Antikörper (ANA), extrahierbare nukleäre Antigene (ENA), CPP(antizyklisches citrulliniertes Peptid)-Antikörper sowie antineutrophile zytoplasmatische Antikörper (ANCA) (Tab. 1), zudem bestand keine humoral entzündliche Reaktion. Bei laborchemisch Hypourikämie mit fehlendem Nachweis von Harnsäure im Serum und im Urin erfolgten ergänzende Stoffwechseluntersuchungen, die massiv erhöhte Xanthinwerte zeigten (Tab. 2).

**Tab. 1:** Rheumatologisches Basis-Labor im Serum

Analyse	Resultat	Referenz
Hämoglobin	144 g/l	120–160
Thrombozyten	190 G/l	150–450
Leukozyten	6,3 G/l	4,5–11,5
Senkung (EDTA)	2 mm/h	< 30
Natrium	138 mmol/l	136–145
Kalium	4,5 mmol/l	3,5–5,1
Calcium	2,31 mmol/l	2,15–2,50
Magnesium	0,88 mmol/l	0,66–1,07
Phosphat	1,24 mmol/l	0,81–1,45
Harnstoff	4,0 mmol/l	2,1–7,1
Kreatinin	60 µmol/l	40–80
eGFR (Krea, CKD-EPI 2009)	106 ml/min/1,73 m^2^	> 60
C‑reaktives Protein	< 0,5 mg/l	< 10
AST (GOT)	29 U/l	5–35
Gamma-GT	10 U/l	< 38
Alkalische Phosphatase	63 U/l	46–116
Harnsäure	< 20 µmol/l	137–357
Vitamin D, 25-OH	57 nmol/l	50–220
CK gesamt	108 U/l	34–145
Parathormon	29,1 ng/l	18,4–80,1
TSH	0,93 mU/l	0,55–4,78
Rheumafaktor	< 4 kIU/l	< 14
CCP IgG	< 0,5 kU/l	< 5
ANA IIF HEp‑2	< 160	< 160

**Tab. 2:** Urinbefund und ergänzende Stoffwechselparameter im Urin

Analyse	Resultat	Referenz
Leukozyten	Negativ	Negativ
Erythrozyten	Negativ	Negativ
Protein	Normal	Normal
Kreatinin	2,4 mmol/l	–
Harnsäure	< 2,7 mmol/mol Kreatinin	229–571
Xanthin	177 mmol/mol Kreatinin	< 5,0
Hypoxanthin	13 mmol/mol Kreatinin	< 13
Inosin	< 0,2 mmol/mol Kreatinin	< 0,8
Dihydrouracil	4,6 mmol/mol Kreatinin	n. d.

**Abb. 1:**
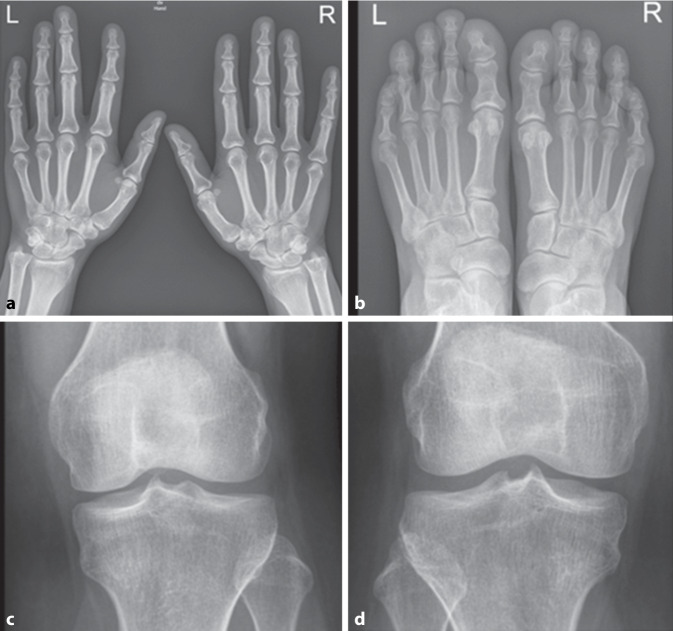
Konventionelle Röntgenaufnahmen der **a** Hände und **b** Füße beidseits mit unspezifischen Veränderungen. An den Knien (**c** *links* und **d** *rechts*) beidseits keine Meniskusverkalkung als Hinweis auf eine Kalziumpyrophosphat-Dihydrat(CPPD)-Arthropathie

**Abb. 2:**
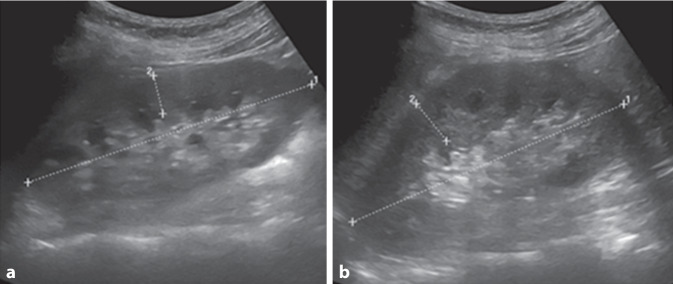
Sonographisch orthotope Lage und regelhafte Größe der Nieren beidseits, **a** r*echts* 11,6 cm lang, **b** *links* 11 cm, mit beidseits regelhafter Parenchymdicke, **a** *rechts* 1,59 cm, **b** *links* 1,91 cm, und kleine Konkremente im Nierenbeckenkelchsystem (Nierengrieß) beidseits ohne Harnstauung

Nativradiologisch bestanden keine typischen arthritischen Veränderungen der Hände und Füße noch Zeichen einer Kristallarthropathie der Knie und Hände (Abb. 1). Bei normwertiger Nierenfunktion und unauffälligem Urinsediment konnten in der Abdomensonographie kleine Konkremente im Nierenbeckenkelchsystem (Nierengrieß) beidseits ohne Harnstauung dargestellt werden (Abb. 2). Zudem ergab sich elektroneurographisch kein Hinweis auf ein Karpaltunnelsyndrom bei ebenfalls unauffälligem Befund der Magnetresonanztomographie der Lendenwirbelsäule.

## Diagnose

Anhand der fehlenden Harnsäure (im Serum und Urin) sowie der erhöhten Xanthin-Spiegel im Urin konnte die Diagnose einer Xanthinurie gestellt werden [1]. Hierbei handelt es sich um eine seltene autosomal-rezessiv vererbte Störung des Purinstoffwechsels, bei der das Enzym Xanthinoxidreduktase (XOR) (Enzym mit beiden Formen Xanthindehydrogenase [XDH] und Xanthinoxidase [XO]) defizitär ist [2].

### Biochemische Eigenschaften, Einteilung und Genetik

**Abb. 3:**
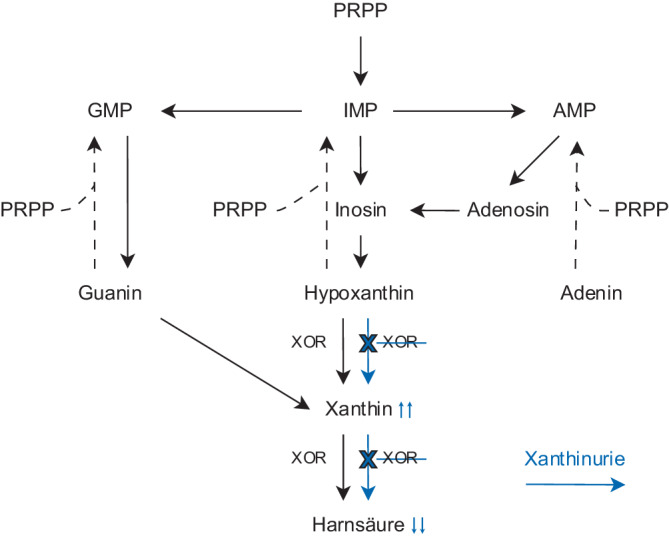
Vereinfachte Darstellung des Purinstoffwechsel physiologisch (*schwarz*) sowie bei Xanthinurie (*blau*): XOR katalysiert die Bildung von Xanthin aus Hypoxanthin und von Harnsäure aus Xanthin. Xanthin wird ebenfalls aus Guanin gebildet. Bei der klassischen Xanthinurie akkumuliert aufgrund der fehlenden Funktion der XOR Hypoxanthin und Xanthin, wobei Hypoxanthin ebenfalls über den Salvage Pathway zurückgebildet wird zu Inosinmonophosphat (IMP)

Die XOR katalysiert die Umwandlung von Hypoxanthin zu Xanthin und von Xanthin zu Harnsäure. Xanthin wird zudem auch aus Guanin gebildet. Die Akkumulation von Hypoxanthin wird größtenteils durch den Salvage Pathway mit 5‑phospho-α-D-ribosyl-1-pyrophosphat (PRPP) als Ko-Substrat in Inosinmonophosphat umgewandelt (Abb. 3; [1, 2]).

Die klassische Xanthinurie wird in 2 Typen eingeteilt während zwischenzeitlich pro Typ mehrere Mutationen beschrieben wurden. Bei Typ 1, in Online Mendelian Inheritance in Man (OMIM), einer medizinischen Online-Datenbank menschlicher Gene sowie genetischer Phänotypen und Erkrankungen als Nummer 278300 bezeichnet, handelt es sich um einen genetischen Defekt der XOR im Chromosom 2p23.1, daher fehlt isoliert die Funktion von XOR. Beim Typ 2 (OMIM 603592) betrifft der genetische Defekt die Molybdän-Kofaktor-Sulfurase (MOCOS) im Chromosom 18q12.2. Diese Mutation verhindert sowohl die Aktivierung der XOR als auch der Aldehydoxidase (AO), was ebenfalls zu einer Akkumulation von Xanthin führt [1, 3, 4].

Der ergänzende Molybdän-Kofaktor-Mangel wird als Xanthinurie Typ 3 (OMIM 252150) bezeichnet, der neben dem Verlust der Enzymaktivität von XOR und AO auch die Sulfitoxidase (SO) betrifft. Da hierbei jedoch betroffene Patienten eine hohe Symptomlast haben (neurologische Ausfälle, Krämpfe, Muskeltonusstörungen, Entwicklungsstörungen, Linsenluxation) und die meisten im ersten Lebensjahr versterben [5, 6], ist dieses in unserem Fall klinisch unwahrscheinlich.

Obwohl die genetischen Defekte verschiedene Gene betreffen, sind die beiden Varianten der klassischen Xanthinurie, Typ I und Typ II, klinisch nicht zu differenzieren [7]. Die Unterscheidung zwischen Typ 1 und Typ 2 kann mit dem Allopurinol-Loading-Test erfolgen, bei dem Oxypurinol (Abbauprodukt aus Allopurinol) im Serum oder Urin nach Verabreichung von Allopurinol gemessen wird. Wird Allopurinol verabreicht, aber nicht zu Oxypurinol metabolisiert, liegt eine Xanthinurie vom Typ 2 vor, bei der sowohl die XOR als auch die AO fehlen, während bei einer Metabolisierung zu Oxypurinol eine Xanthinurie vom Typ 1 vorliegt [1]. Die Diagnose einer Xanthinurie kann zudem durch eine niedrige XDH-Aktivität in der Leber oder der Duodenalschleimhaut mittels Biopsie bestätigt werden [3, 8]. Klinisch ist dies jedoch nicht relevant, da die Therapie und Prognose sich nicht zwischen beiden Xanthinurietypen unterscheiden.

Die Xanthinurie wird autosomal-rezessiv vererbt [1]. Die Großmütter unserer Patientin waren Cousinen, was eine Wahrscheinlichkeit einer Homozygote bei Nachkömmlingen erhöht. Die erweiterte Familienanamnese ergab ebenfalls Nierensteinleiden bei Verwandten (Vater und Großvater väterlicherseits sowie mütterlicherseits). Lebende Verwandte (2 Brüder, die Schwester und 3 Kinder unserer Patientin) sind bisher asymptomatisch mit normwertiger Harnsäure im Serum. Eine genetische Untersuchung wurde nicht durchgeführt.

### Epidemiologie und Literatur-Review

**Abb. 4:**
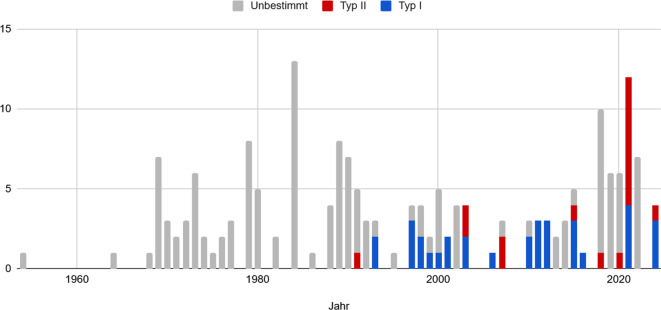
Publizierte Fallberichte der Xanthinurie im zeitlichen Verlauf bis 2024 (*blau* Typ I vs. *rot* Typ II vs. *grau* unbestimmt)

Der erste Fall einer Xanthinurie wurde von Dent und Philpot im Jahr 1954 beschrieben [9]. Weltweit sind bisher nur wenige Fallberichte von Patienten mit Xanthinurie veröffentlicht. Die Inzidenzschätzung liegt zwischen 1:100.000 und 1:1.000.000, aber manche Autoren berichten, dass die Erkrankung stark unterdiagnostiziert sei [10]. Eine systematische Literaturrecherche aller Fallberichte in PubMed ergab 104 Originalarbeiten mit 186 Patienten (s. elektronisches Zusatzmaterial online). Hierbei wird oft nicht sicher zwischen Xanthinurie Typ 1 und 2 differenziert (Abb. 4). Weniger frühe veröffentlichte Fallberichte spezifizieren zudem nicht, ob es sich um eine Neubeschreibung oder ein Follow-up handelt. Die Anzahl der veröffentlichten Fälle zeigt jedoch über die Jahre keine Veränderung.

## Symptome

Die meisten Patienten mit Xanthinurie Typ I und II sind asymptomatisch, mögliche klinische Symptome sind jedoch Urolithiasis aufgrund erhöhter Xanthinausscheidung und Myalgien aufgrund von intramuskulären Xanthinablagerungen. Als weitere Komplikationen wurden Arthralgien und Niereninsuffizienz im Verlauf der Erkrankung beschrieben [1, 11, 12]. Klinisch stehen in unserem Fall neben der Nephrolithiasis die typischen Symptome der „Fibromyalgie“ (chronische generalisierte muskuloskeletale Schmerzen, Fatiguesymptomatik, Schlafstörungen sowie kognitive Einschränkungen und depressive Symptomatik [13–16]) im Vordergrund, die sich teilweise mit den möglichen Symptomen einer Xanthinurie überlappen (Tab. 3). Es kann klinisch nicht sicher differenziert werden, ob es sich bei den Symptomen um 2 unabhängige Erkrankungen handelt oder um eine sekundäre Fibromyalgie als Folge der Xanthinurie.

**Tab. 3:** Typische Symptome Fibromyalgie [13–16] und Xanthinurie [1, 11, 12]

Fibromyalgie (%)	Xanthinurie (%)
Myalgien (~ 45)	Myalgien (~ 6,9)
Arthralgien (~ 45)	Arthralgien (~ 5,1)
Fatigue (~ 40 %)	Urolithiasis (~ 30)
Kognitionsstörung (~ 40)	–
Depression (~ 40)	–
Schlafstörung (~ 30)	–

## Therapie und Verlauf

Xanthin ist in Wasser schwer löslich, und die Löslichkeit wird durch den pH-Wert nur geringfügig beeinflusst, sodass eine Alkalisierung des Urins nicht wirksam ist, um die Steinbildung zu verhindern. Die einzige Prophylaxe besteht in einer erhöhten Diurese (vermehrte Dilutation durch gesteigerte Flüssigkeitszufuhr) und einer purinarmen Diät (verminderte Steinbildung durch Substratmangel). Kontraintuitiv gleichen sich somit die alimentäre Therapie der Hypo- und Hyperurikämie. Im Verlauf ist insbesondere die interdisziplinäre nephrologische Mitbetreuung notwendig, da die Entwicklung einer Niereninsuffizienz möglich ist [17–20].

## Fazit für die Praxis

Auffälligen Befunden sollte auch dann nachgegangen werden, wenn eine andere Erkrankung wie Fibromyalgie ausreichend diagnostiziert werden kann. Bei Patienten mit unklarer Hypourikämie sind detaillierte Untersuchungen der Purinstoffwechselparameter erforderlich, da eine frühzeitige Diagnose und Behandlung mit hoher Flüssigkeitszufuhr und Ernährungsumstellung Komplikationen wie Nierensteine reduzieren können.

## Supplementary Information

Systematische Literatur-Recherche aller Fallberichte in PubMed
